# Ileal Duplication Cyst Causing Recurrent Abdominal Pain and Melena

**Published:** 2010-08-14

**Authors:** Muhammad Ali Sheikh, Tariq Latif, Masoom Ali Shah, Imran Hashim, Akhtar Jameel

**Affiliations:** Department of Pediatric Surgery, Shaikh Zayed Hospital Lahore, Pakistan

**Keywords:** Alimentary tract duplications, Pain abdomen, Melena

## Abstract

Alimentary tract duplications are rare congenital anomalies. The presentation depends on their anatomical location, size and other characteristics. The most common variety is small bowel cystic duplication. We report a case of an eight years old girl who presented with recurrent abdominal pain and melena. Radioisotope technetium scan showed increased uptake of tracer in right lower abdomen and a diagnosis of Meckel’s diverticulum made. At surgery a cystic, communicating, ileal duplication found which was resected along with adjacent gut. It is thus reiterated that while investigating children with recurrent abdominal pain and melena, gut duplications must be included in the differential diagnosis.

## INTRODUCTION

Alimentary tract duplications (ATD) are rare congenital anomalies [1]. These lesions may be present anywhere from mouth to anus but commonly found in small bowel [2]. The reported incidence of these lesions is 1 in 4500 [3]. In 1937, Ladd coined the term “duplications of the alimentary tract” thus simplifying the terminology which previously used to be confusing [4]. The term is applied to those lesions that had well developed coat of smooth muscle, mucosa representing some part of alimentary tract with close anatomic relation with some portion of gastrointestinal tract.

Duplications of small bowel can cause life threatening complications like volvulus, intussusception, gut perforation and massive bleeding. Herein we report one such case that remained elusive till laparotomy was performed.

## CASE REPORT

An eight years old girl was admitted in medical ward through emergency department because of recurrent abdominal pain and melena for the last one month. She had similar complaints since the age of 3 years. The symptoms usually settled within few days. At the time of admission she was otherwise healthy but pale looking with tenderness around umbilicus. Her weight was 19 kg, hemoglobin 6.8 gram per cent and hematocrit 21 percent. Her coagulation profile was within normal limits. Abdominal radiograph and ultrasound examination were reported as insignificant.

Radioisotope technetium scan done for suspected Meckel’s diverticulum showed increased tracer uptake in right lower abdomen close to urinary bladder suggestive of ectopic gastric mucosa (Fig. 1). She was prepared for surgery with transfusion of packed cells and initially underwent laparoscopy. The lesion could not be localized so laparotomy was performed. The omentum was found adhered to a lesion in the mid ileum and gut was twisted around it. On separation about 4×5 centimeter cystic structure found in the mesentery of ileum two feet proximal to the cecum (Fig. 2). The cystic structure with adjacent part of ileum was resected and continuity of gut restored with a primary anastomosis. The duplicated part was found communicating with both proximal and distal parts of gut resected. The post-operative recovery was uneventful. Histology of resected gut confirmed the presence of gastric mucosa in duplicated part. 

**Figure:**
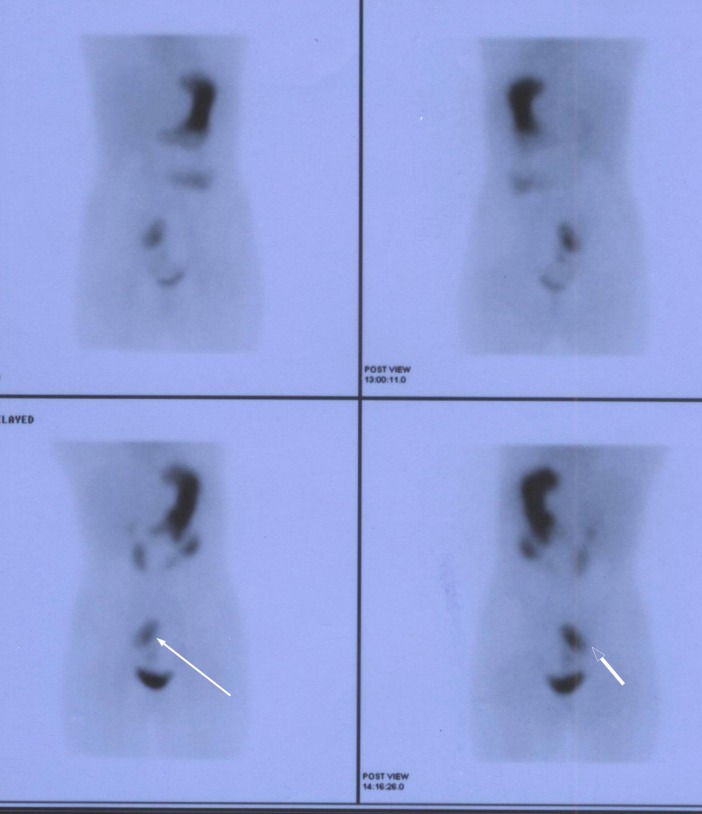
Figure 1: Radioisotope scans showing abnormal uptake in lower abdomen (white arrow).

**Figure:**
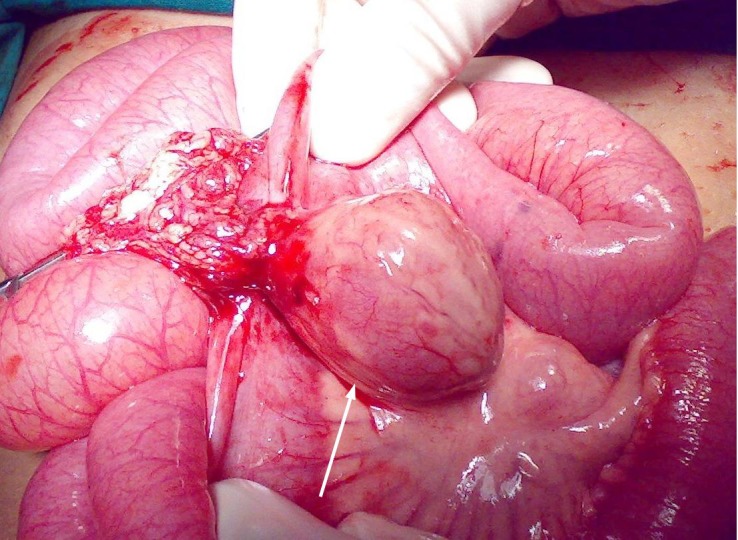
Figure 2: Cystic duplication of ileum (white arrow).

## DISCUSSION

Alimentary tract duplication cysts are hollow, epithelium-lined, cystic, spherical or tubular structures that are attached to the wall of gastrointestinal tract [4]. They may or may not communicate with the intestinal tract. Different theories had been put forward to explain the occurrence of enteric duplications but no single theory could account for all the known variants. The split notochord theory was postulated to explain the formation of neuroenteric duplications and associated vertebral anomalies [4 , 5 , 6]. Some duplications of foregut and hindgut may result from partial twinning. Others especially of ileum, may occur as a result of persistent embryological diverticula. Some portions of the alimentary tract undergo a solid stage during development followed by recanalization; therefore duplications may result from aberrant luminal recanalization [4]. Finally intrauterine insults such as trauma or hypoxia could cause these anomalies at any level of alimentary tract thus etiology may be multi-factorial. Duplications may be found along esophagus in thorax, mid gut and in hindgut with involvement of urinary and genital system [7]. The most common location is ileum and majority are cystic in nature [2 , 6]. More than 50% of the cystic duplications in all locations have gastric mucosa. In our patient the lesion was of cystic variety with ectopic gastric mucosa.

The clinical presentation of these lesions can vary according to the age of the patient as well as anatomical location. Some may remain asymptomatic and identified on routine physical examination or during investigations for other problems [2]. The small bowel ATD can be an anchor point for intussusceptions or may result in volvulus, whereas long tubular duplications with proximal communication drain poorly and retention of intestinal contents can obstruct adjacent intestine. Gastric mucosa in duplication can cause ulceration, bleeding or perforation. These duplications can mimic inflammatory bowel diseases as documented by Puligandla, because six of their patients were treated as atypical Crohn’s disease [2]. The widespread use of prenatal ultrasound scan has allowed these lesions to be detected early in gestation. With prenatal diagnosis treatment can be instituted before the onset of symptoms. Laparoscopy can be used as a diagnostic modality in cases of recurrent abdominal pain.

The diagnosis is usually not established before surgery as occurred in our patient where Meckel’s diverticulum was suspected based upon radioisotope findings thus duplications must be considered in differential diagnosis of recurrent abdominal pain and occult gastro intestinal bleeding. Cystic duplications can be resected easily along with adjacent intestine and same was possible in case reported here. Presence of adhered omentum at surgery points towards episodes of inflammation of the cyst. Nowadays laparoscopy is gaining importance in managing different pediatric surgical conditions. This minimally invasive procedure was used in this patient but had to be converted to open approach, as the lesion could not be identified. With increasing learn curve we hope to use this technique more liberally.

## Footnotes

**Source of Support:** Nil

**Conflict of Interest:** None declared
